# The Anti-Fasciolasis Properties of Silver Nanoparticles Produced by *Trichoderma harzianum* and Their Improvement of the Anti-Fasciolasis Drug Triclabendazole

**DOI:** 10.3390/ijms141121887

**Published:** 2013-11-05

**Authors:** Youssuf A. Gherbawy, Ismail M. Shalaby, Mahmoud Syed Abd El-sadek, Hesham M. Elhariry, AbdelElah A. Banaja

**Affiliations:** 1 Department of Biological Sciences, Faculty of Science, Taif University, P.O. Box 888, Taif 21974, Saudi Arabia; E-Mails: ismail.mehrez@gmail.com (I.M.S.); hesham_elhariry@yahoo.com (H.M.E.); aabanaja@yahoo.com (B.A.A.); 2 Botany Department, Faculty of Science, South Valley University, Qena 83523, Egypt; 3 Nanomaterial Lab., Physics Department, Faculty of Science, South Valley University, Qena 83523, Egypt; E-Mail: mahmoud.abdelsadek@sci.svu.edu.eg; 4 Department of Food Science, Faculty of Agriculture, Ain Shams University, POB 68- Hadayek Shoubra, Cairo 11241, Egypt

**Keywords:** egg hatching, *Fasciola*, silver nanoparticle, Triclabendazole, *Trichoderma*

## Abstract

Recently, new strains of *Fasciola* demonstrated drug resistance, which increased the need for new drugs or improvement of the present drugs. Nanotechnology is expected to open some new opportunities to fight and prevent diseases using an atomic scale tailoring of materials. The ability to uncover the structure and function of biosystems at the nanoscale, stimulates research leading to improvement in biology, biotechnology, medicine and healthcare. The size of nanomaterials is similar to that of most biological molecules and structures; therefore, nanomaterials can be useful for both *in vivo* and *in vitro* biomedical research and applications. Therefore, this work aimed to isolate fungal strains from Taif soil samples, which have the ability to synthesize silver nanoparticles. The fungus *Trichoderma harzianum*, when challenged with silver nitrate solution, accumulated silver nanoparticles (AgNBs) on the surface of its cell wall in 72 h. These nanoparticles, dislodged by ultrasonication, showed an absorption peak at 420 nm in a UV-visible spectrum, corresponding to the plasmon resonance of silver nanoparticles. The transmission electron micrographs of dislodged nanoparticles in aqueous solution showed the production of reasonably monodisperse silver nanoparticles (average particle size: 4.66 nm) by the fungus. The percentage of non hatching eggs treated with the Triclabendazole drug was 69.67%, while this percentage increased to 89.67% in combination with drug and AgNPs.

## 1. Introduction

*Fasciola* spesies is an important parasite of sheep and camel, so it has been the subject of many scientific investigations. This is not only because of its high prevalence rates but also due to its enormous production losses in these animals reported from various parts of the world. In addition, fasciolosis is now recognized as an emerging human disease by the World Health Organization. Fascioliasis, a serious infectious parasitic disease infecting domestic ruminants and humans, tops all the zoonotic helminthes worldwide [1]. It is caused by a liver fluke belonging to genus *Fasciola*. A large variety of animals, such as sheep, goats, cattle, buffalo, horses, donkeys, camels and rabbits, show infection rates that may reach 90% in some areas [2]. The infection with *Fasciola* spp. represents a major human health problem in diverse parts of Africa such as Egypt, Zambia, Kenya, Algeria, Zimbabwe, Tanzania and Nigeria [3–10], and recently, human infection cases with *F. hepatica* have been documented from southwest Tunisia, with prevalence infection of 6.6% [11]. In Saudi Arabia, *Fasciola gigantic* counted as one of the most common gastrointestinal helminthes in camels in Saudi Arabia [12]. *Fasciola* sp. were with 14.5% prevalence from leafy vegetables in Riyadh region [13]. Fascioliasis among local and imported sheep in Saudi Arabia was studied [14]. This study showed that, the detection of eggs revealed 13.5% infection rate compared with 21.9% by detection of worms (*p* < 0.001). In addition, the infection rate was significantly higher (*p* < 0.001) among the imported sheep (15.1%) than among the local ones (4.96%). A report on stool analysis of male immigrant manual workers in Saudi Arabia, suggested that nine out of ten patients suffered from human fascioliasis [3,14,15]. Abou-Zinadah and Fouad [16] examined 20 imported sheep for fascioliasis natural infection by kato thick smear and by Fasciola-indirect haemagglutination test (IHAT). They reported that stool examination revealed infection in 13/20 (65%), but IHAT identified 11/20 (55%). Therefore, the sensitivity was 84.5%.

At present, there is no vaccine available for the prevention of fascioliasis [17], and hence chemotherapy is the current mainstay of control. Triclabendazole is the drug of choice as it is safe and efficacious against both juvenile and adult flukes [18]. There is considerable concern of drug resistance development in humans, as drug resistance has already been reported from sheep and cattle from different parts of the world as e.g., Australia, Ireland, The Netherlands, Spain and the UK [19,20]. Currently, the interest in nanotechnological approaches in microbiology is growing rapidly, as in all fields of the science [21,22]. The main motive is the expectation that nanoparticles will be able to be used in the treatment of various diseases in the future [23–25]. In recent studies, it was determined that through their unique properties and large surface areas, metal oxide nanoparticles possess effective antimicrobial activities [25]. Particularly, owing to their great chemical reactivity, nanoparticles are capable of producing reactive oxygen species (ROS), which have the ability to kill infectious agents. The antibacterial and antiviral behaviors of silver, silver ions, and silver-containing compounds have long been investigated [26,27]. Ag-NPs demonstrated significant antileishmanial effects by inhibiting the proliferation and metabolic activity of promastigotes by 1.5- to threefold, respectively, in the dark, and 2- to 6.5-fold, respectively, under UV light [28]. The preparation of uniform nanosized silver particles with specific requirements in terms of size, shape, and physical and chemical properties is of great interest in the formulation of new pharmaceutical products [29,30]. Currently, there is a growing need to using environmentally friendly nanoparticles that do not produce toxic wastes in their process synthesis protocol. To achieve this, we are inclined to shift to benign synthesis processes, which happen to be mostly of biological nature [31].

In the present investigation, we explore the potential of *Trichoderma harzianum* to synthesize AgNPs and test the drug efficacy of Triclabendazole alone and with AgNPs on egg hatching of *Fasciola* eggs.

## 2. Results and Discussion

**Figure 1:**
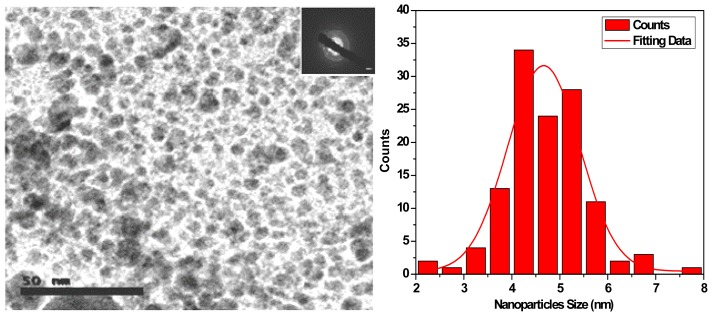
HRTEM image and size dispersion histogram of Ag nanoparticles.

HRTEM was employed to obtain direct information about the size of the produced Ag nanoparticles. Figure 1 shows the HRTEM image and the distribution size diagram of the Ag nanoparticles. The particles were separately dispersed on a on a carbon coated copper TEM grid and transmission electron microscopy (TEM) investigations were analyzed. TEM image of Ag nanoparticles revealed crystalline, free-standing, and approximately spherical particles as well as a number of aggregates. Average particle size calculated by log-normal fitting to the size distribution histogram, was obtained as 4.66 nm. The silver nanoparticles are crystalline, as can be seen from the selected area diffraction pattern recorded from one of the nanoparticles in the aggregates (Figure 1 Inside). The separation between the silver nanoparticles seen in the TEM image could be due to capping by proteins and would explain the UV-Vis spectroscopy measurements, which is characteristic of well-dispersed silver nanoparticles. These monodispersed silver nanoparticles (biosynthesized by *Trichoderma viride*) showed emission in the range of 320–520 nm wavelength [32]. Considering the UV-vis intensity, wavelength, TEM and particle size distribution the most promising results obtained indicate that, the optimum conditions for preparation of silver nanoparticles colloids with excellent size and size distribution ranged from 3 to 8 nm could be produced using 10 g biomass of fungus *F. solani*; 0.085 g AgNO_3_; pH 12; temperature, ~25 °C and duration, 48 h [33]. The morphology of the nanoparticles synthesized by *Trichoderma reesei* was highly variable and these assemblies were found to be aggregates of silver nanoparticles in the size range 5–50 nm [34]. In addition, the nanoparticles were not in direct contact even within the aggregates, indicating stabilization of the nanoparticles by a capping agent [34]. AgNPs synthesized by different species of *Trichoderma* were found single or aggregated with round and uniform in shape and 6–80 nm in size [35]. Efficacy of AgNPs biosynthesis was reported due to reductase action or by electron shuttle quinones or both [36]. In fact, it is shown that the presence of hydrogenase and nitrate reductase are essential elements for metal reduction [36,37].

**Figure 2:**
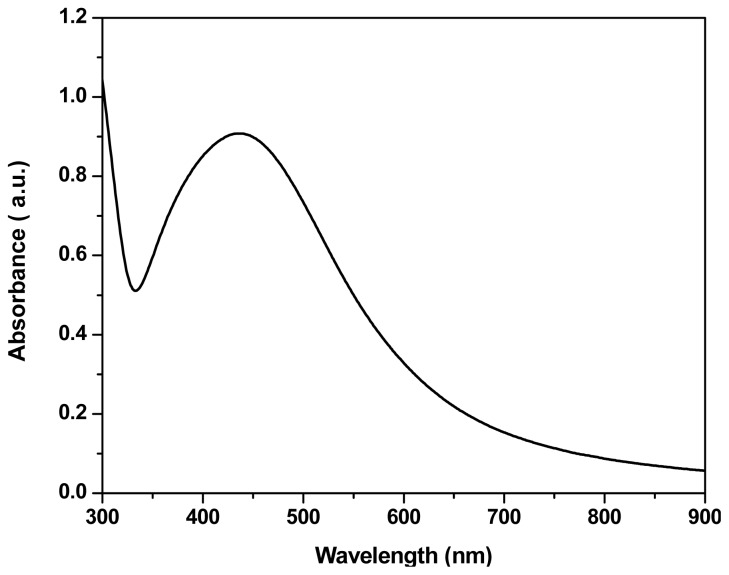
UV-visible spectra of fungal filtrate containing Ag nanoparticles.

UV-visible absorption spectra for Ag NPs prepared from *Trichoderma harzianum* using silver nitrate are shown in Figure 2. The spectra recorded from the Ag NPs solution, it is seen that a blue shift in the onset of absorption is observed in this sample and showed an absorbance peak at 420 nm, which was specific for the silver nanoparticles [38].

This phenomenon of blue shift of absorption edge has been ascribed to a decrease in particle size. This results in a shift in the absorption edge to a lower wavelength region. Furthermore, nanoparticles are highly sensitive and functionally efficient because of smaller grain size and high surface to volume ratio as compared to the conventional materials in micrometer range where in case of nano-scale materials most atoms, ions and defects would be on the surface.

It is observed, from the spectra of *Trichoderma viride* synthesized silver nano solution, that the silver surface plasmon band occurs at 405 nm in addition to prominent band at around 260 nm [32]. *Aspergillus flavus* when challenged with silver nitrate solution a characteristic surface Plasmon absorption band at 420 nm was observed at 24 h that attained the maximum intensity after 72 h. After 72 h of incubation, no change in intensity at 420 nm was observed indicating complete reduction of silver ions [39]. The ultraviolet-visible spectra of *Aspergillus terreus* cell filtrate with AgNO_3_ showed a strong broad peak at 440 nm (SPR band), which indicated the presence of AgNPs [40]. Devi *et al*. [35] screened 75 isolates belonged to 5 *Trichoderma* species for Ag NBs. Their results indicated that the high Plasmon band was observed at 420 nm at every 24 h.

Previously studies reported that the antimicrobial activities of AgNPs are size-dependent, the smallest sized showed the strongest effect [41]. Therefore, we already tested the smallest size of AgNPs (4.66 mm). Table 1 shows the results of *Fasiola* egg hatching experiment. The table shows that eggs were hatched at a percentage of 100% in non-treated control eggs. As for the *in vitro* treated eggs with the drug alone the ratio was 29.4% hatched and 70.6% non-hatched. As for that treated with the drug together with the silver nano particles (biologically synthesized from *Trichoderma harzianum*) the ratio was 9.4% hatched and 90.6% non-hatched. Applying ANOVA test on the hatched and non-hatched data with both treatments showed that *p* < 0.001 (highly significant), with *F* value 96.257.

**Figure 3:**
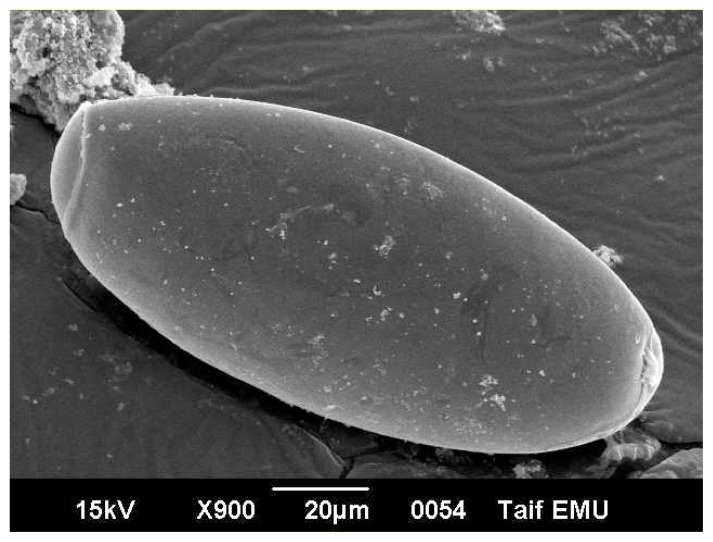
*Fasciola* egg (control, non treated) Notice the smooth texture of the egg surface.

**Figure 4:**
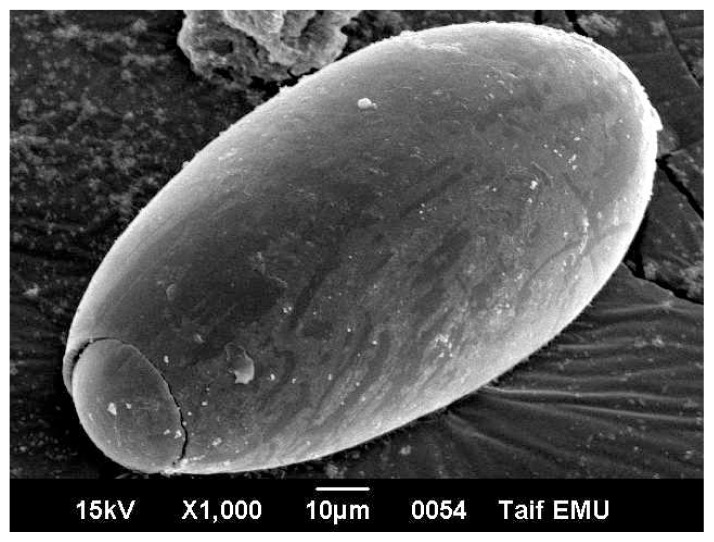
*Fasciola* egg treated *in vitro* with the drug only. Notice the alterations on the egg surface due to drug administration.

**Figure 5:**
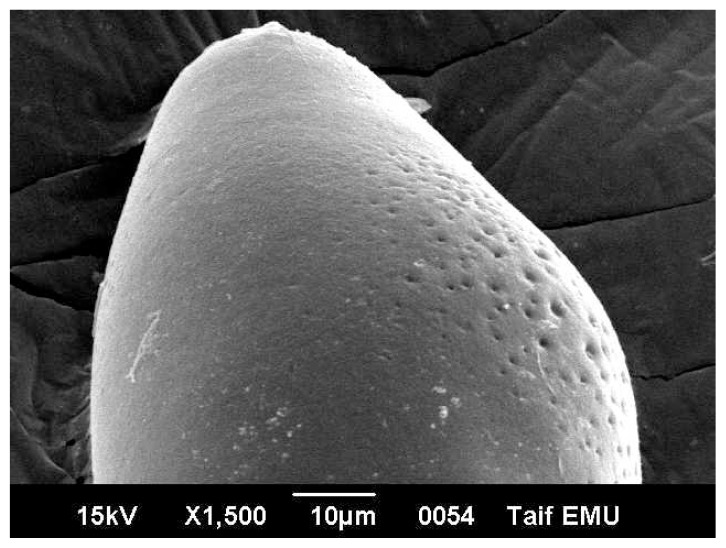
*Fasciola* egg treated *in vitro* with the drug with nano silver particles. Notice the perforations on the egg surface due to the effect of drug carried on nano silver particles.

Egg of *Fasciola* is oval measuring 120 micron long and 40 micron wide. It has an operculum on its top. The egg is immature when first laid by the worm. Under electron microscope, the egg surface texture is smooth with no ornamentations (Figure 3). Electron micrographs of eggs representing the three patches are showed in Figures 3–5. Figure 3 shows non-treated control egg, notice the smooth texture of the egg shell and the non-distorted eggs. Figure 4 shows the egg treated with the drug alone, notice the partly distorted surface and rough texture of the egg surface. Figure 5 shows the egg treated with the drug with the silver nano particles, notice the more distorted surface and the perforations that are obviously shown on the egg surface.

The results in Table 1 indicated that 30.33% of *Fasciola* eggs hatched in the presence of Triclabendazole sulfoxide (TCBZ). The results of Moll *et al*. [42] showed a significant reduction of 99.7%, 98.1% and 99.2%, respectively, in fluke egg output at 21 days in all non-TCBZ treated animals. In addition, they reported that, TCBZ treatment produced percentage decreases of 15.3%, 4.3% and 36.6%, respectively. These results are highly indicative of the presence of TCBZ-resistant *Fasciola hepatica* in sheep and cattle on this farm. The efficacy of the drug has been reported to be minimized due to drug resistance in animals [43,44]. The results of the present work showed that the drug efficacy on the hatchability of *Fasciola* eggs is increases drastically using silver nano particles combined with the drug of choice (10.33% and 30.33% respectively). Triclabendazole (TCBZ) is inhibiting of microtubule-dependent processes as a result of binding to the β-tubulin molecule [45–47]. However, if TCBZ action is associated with microtubule depolymerisation, some egg damage would be expected considering that the drug may accumulate within the egg in therapeutic concentrations [48]. Figure 5 showed the penetration sites of AgNPs to egg shill. This AgNPs may be cause membrane damage. The NPs that anchor onto *Escherichia coli* cell surface and penetrating the cells may cause membrane damage, which could result in cell lysis [49]. Ciprofloxacin-loaded nanoparticles were more active in human macrophages infected with *Mycobacterium avium* complex than free drug [50]. The efficacy of ampicillin was found to be increased by 120-fold in murine salmonellosis by loading the drug into the nanoparticles [51]. The azithromycin nanoparticle preparations showed appropriate physicochemical and improved antimicrobial properties, which can be useful for oral administration [52]. Efficacy and broadening of antibacterial action of drugs enhanced via the use of capped Mesoporous nanoparticles [53]. In addition, surface plasmon enhanced drug efficacy using core-shell Au@SiO_2_ nanoparticle carrier as reported recently [54].

## 3. Materials and Methods

### 3.1. Source of Microorganisms

The fungus culture, *Trichoderma harzianum* (TUT89) was isolated from soil sample In Taif region and maintained in potato dextrose agar (HiMedia, Mumbai, India) slant at 27 °C. Internal transcribed spacer region (ITS) sequence for this strain was deposited in GenBank and its accession number is HE649481.

### 3.2. Production of Biomass

To prepare the biomass for biosynthesis studies the fungus was grown aerobically in liquid broth containing (g/L) KH_2_PO_4_, 7; K_2_HPO_4_, 2; MgSO_4_·7H_2_O, 0.1; (NH_2_)SO_4_, 1; yeast extract, 0.6; glucose, 10. The culture flasks were incubated on an orbital shaker at 27 °C and agitated at 150 rpm. The biomass was harvested after 72 h of growth by sieving through a plastic sieve followed by extensive washing with sterile double-distilled water to remove any medium components from the biomass [32].

### 3.3. Synthesis of AgNPs

Typically 20 g of biomass (wet weight) were brought into contact with 100 mL sterile double-distilled water for 48 h at 27 °C in an Erlenmeyer flask and agitated as described above. After incubation, the cell filtrate was filtered by Whatman filter paper No.1 (Oakland, CA, USA). After filtration, the observed pH of cell filtrate was 7.2. Into these 100 mL of cell filtrate, a carefully weighed quantity of silver nitrate was added to the Erlenmeyer flask to yield overall Ag+ ions concentration of 10–3 M, and the reaction was carried out under dark conditions [32].

### 3.4. Characterization of AgNPs

#### 3.4.1. UV-Visible Spectral Analysis

Change in color observed in the silver nitrate solution incubated with the tested strains. The UV-visible spectra of this solution recorded in spectrophotometer, from 200 to 800 nm, at an interval of 24 h. The silver nanoparticles dispersed in water by ultrasonication will be kept at room temperature (37 °C) for 3 months and the absorption peak at 420 nm will be measured continuously, to determine their stability.

#### 3.4.2. SEM, TEM and Electron Diffraction Analysis

The freeze-dried mycelial mats (positive control and silver nitrate treated sample) mounted on specimen stubs with double-sided adhesive tape and coated with gold/palladium in a sputter coater and examined under SEM at 12–15 kV with a tilt angle of 45°. For TEM, a drop of aqueous solution containing the silver nanomaterials placed on the carbon coated copper grids and dried under Infrared lamp.

#### 3.4.3. Effect of Triclabendazole and Ag NPs in *F. hepatica*

Six-well multidishes were used for the experiments. One hundred eggs of *F. hepatica* isolate were placed into each well, as much water as possible drawn off, then 3 mL of a Triclabendazole sulfoxide (TCBZ.SO) solution (a drug of choice for Fascioliasis), specific concentration of AgNPs (50 μg/mL) and combination of Ag-NPs with TCBZ was added at a specific concentration (0.1 μg/mL); this was also true of the dimethyl sulfoxide (DMSO) solution in the control well. One replicate of each was set up in the same multidish, together with a separate control well containing DMSO at a concentration of 0.5% (*v*/*v*) in tap water. Previous experiments had shown that, at this concentration, the solvent did not affect egg development and hatching [55]. Separate multidishes were set up in the same way, to give a minimum of 3 replicates in total. The multidishes were placed into a dark incubator at 25° and monitored every other day over a period of 14 days. At the end of the 14-day time period, the drug solution in each well was carefully removed and replaced with 3 mL of tap water. The plates were placed on a light box for 1–2 h to stimulate hatching, then each well was examined under a dissecting microscope and the number of eggs at each stage of development recorded using a tally chart [55].

#### 3.4.4. Data Processing and Statistics

All experiments were repeated at least three times in triplicate wells. The results were expressed as the mean plus or minus the standard deviation. Data were analyzed using SPSS software (version 16.0 for Windows; SPSS Inc, Chicago, IL, USA) and *p* < 0.05 values were considered statistically significant.

## 4. Conclusions

The present study indicates Ag-NPs has considerable antifasciolasis activity comparison with Triclabendazole drug, so deserving further investigation for clinical applications.

## Figures and Tables

**Table 1:** A comparison between hatched and non-hatched *Fasciola* eggs in both treatments (Drug alone) and (drug with AgNPs). As between both treatments the data is almost the same (*p* value is <0.001).

Number of eggs	Non-treated eggs		Eggs *in-vitro* treated with the drug alone		Eggs *in-vitro* treated with the drug combined with nano particles	

	hatched	non-hatched	hatched	non-hatched	hatched	non-hatched
100	100	0	30	70	10	90
100	100	0	25	75	5	95
100	100	0	32	68	8	92
100	100	0	28	72	11	89
100	100	0	35	65	15	85
100	100	0	32	68	13	87
Mean	100	0	30.33	69.67	10.33	89.67
SD	0	0	3.50	3.50	3.56	3.56
*F* values	0	0	96.26		96.26	
Significance	0	0	<0.001		<0.001	
Percentage	100%	0%	30.33%	69.67%	10.33%	89.67%
